# Naturally Occurring Acetylcholinesterase Inhibitors and Their Potential Use for Alzheimer's Disease Therapy

**DOI:** 10.3389/fphar.2018.01192

**Published:** 2018-10-18

**Authors:** Thaiane Coelho dos Santos, Thaís Mota Gomes, Bruno Araújo Serra Pinto, Adriana Leandro Camara, Antonio Marcus de Andrade Paes

**Affiliations:** ^1^Laboratory of Experimental Physiology, Department of Physiological Sciences, Biological and Health Sciences Centre, Federal University of Maranhão, São Luís, Brazil; ^2^Health Sciences Graduate Program, Biological and Health Sciences Centre, Federal University of Maranhão, São Luís, Brazil

**Keywords:** Alzheimer's disease, acetylcholinesterase inhibitors, anti-cholinesterase, plant species, secondary metabolites

## Abstract

Alzheimer's disease (AD) is a main cause of dementia, accounting for up to 75% of all dementia cases. Pathophysiological processes described for AD progression involve neurons and synapses degeneration, mainly characterized by cholinergic impairment. This feature makes acetylcholinesterase inhibitors (AChEi) the main class of drugs currently used for the treatment of AD dementia phase, among which galantamine is the only naturally occurring substance. However, several plant species producing diverse classes of alkaloids, coumarins, terpenes, and polyphenols have been assessed for their anti-AChE activity, becoming potential candidates for new anti-AD drugs. Therefore, this mini-review aimed to recapitulate last decade studies on the anti-AChE activity of plant species, their respective extracts, as well as isolated compounds. The anti-AChE activity of extracts prepared from 54 plant species pertaining 29 families, as well as 36 isolated compounds were classified and discussed according to their anti-AChE pharmacological potency to highlight the most prominent ones. Besides, relevant limitations, such as proper antioxidant assessment, and scarcity of toxicological and clinical studies were also discussed in order to help researchers out with the bioprospection of potentially new AChEi.

## Introduction

Alzheimer's disease (AD) is a main cause of dementia, accounting for up to 75% of all dementia cases and has become a population aging-related concern for policymakers and public health systems around the world by its both direct and indirect costs (Takizawa et al., 2015; Fiest et al., 2016; Scheltens et al., 2016). Nowadays, AD prevalence among people over 60 years old is estimated in 40.2 per 1000, while its incidence proportion is 34.1 per 1,000 (Prince, 2015; Fiest et al., 2016). Those values mean that over 45 million people is suffering from AD symptoms worldwide, whereas this scenario is expected to double every 20 years until at least 2050 (Scheltens et al., 2016). AD is mainly characterized by progressive neurodegenerative disorder, clinically demonstrated by cognitive and memory decline, progressive impairment of daily activities, and a variety of neuropsychiatric symptoms and behavioral disturbances (Tarawneh and Holtzman, 2012).

Pathophysiological processes described for AD progression involve neurons and synapses degeneration resulting from beta-amyloid (Aβ) protein aggregation and neurofibrillary tangles, as well as, neuroinflammation, mitochondrial damage, oxidative stress and excitotoxicity, which interfere with several neurotransmitters signaling pathways (Madeo and Elsayad, 2013; Godyn et al., 2016; Henstridge et al., 2016). Among the latter, cholinergic dysfunction is the most studied and has been closely associated with the early cognitive decline found in AD patients (Craig et al., 2011). In fact, early in the 70's, it was observed that cholinergic neurons were prematurely lost in AD process, arising the Alzheimer's Cholinergic Hypothesis (Bartus et al., 1982). This hypothesis was further corroborated by observations that cholinergic neurons in basal forebrain are severely damaged during AD progression (Bartus, 2000).

Despite the huge research on AD, supportive care from family and other caregivers is still the mainstay treatment, though pharmacotherapy has importantly evolved during the last decade. Four drugs are currently used for the treatment of the dementia phase: the acetylcholinesterase (AChE) inhibitors (AChEi)–donepezil, rivastigmine, and galantamine–and the glutamate antagonist memantine. AChEi increase synaptic acetylcholine (ACh) levels and improve cholinergic function in the brain (Anand and Singh, 2013; Andrieu et al., 2015). Amongst those clinically relevant AChEi, galantamine is the only naturally occurring substance, consisting of an alkaloid extracted from Amaryllidaceae family (Heinrich, 2010; Murray et al., 2013). Galantamine reversibly and competitively inhibits AChE (Thomsen and Kewitz, 1990) and allosterically modulates nicotinic ACh receptors (Schrattenholz et al., 1996). Notwithstanding, besides its anti-AChE activity, most of natural AChEi molecules generally present additional pharmacological properties, particularly antioxidant, which enable them to be applied as multi-target strategies against AD onset and progression (Orhan et al., 2011; Ayaz et al., 2017; Sahoo et al., 2018).

Several studies have been carried out toward identification and isolation of natural molecules applicable for design and development of new anti-AD drugs, particularly those pertaining to the classes of alkaloids, terpenes, coumarins and polyphenols (Huang et al., 2013). Therefore, this mini-review recapitulates last decade studies on the anti-AchE activity of plant species, their respective extracts, as well as isolated compounds, in order to settle down the state-of-the-art in the field and to help researchers out with the bioprospection of potentially new AChEi candidates applicable for anti-AD drug design and pharmacotherapy.

## Methodology

This mini-review revises published studies available in Pubmed between 2007 and 2018 (1st semester), which were retrieved by using the following descriptors combination: “anti-acetylcholinesterase and plant extract” and “acetylcholinesterase inhibitors and plant extract and Alzheimer.” The only criterion for inclusion was that anti-AChE activity of the plant extract and/or isolated compounds had been assessed by Ellman's methodology (Ellman et al., 1961), which is considered a gold standard for AChEi screening (Holas et al., 2012). On the other hand, two criteria for exclusion were applied: the lack of reliable positive controls, which might include but are not limited to galantamine, huperzine A and B, or physostigmine (Mehta et al., 2012); and the absence of half maximal inhibitory concentration (IC_50_) assessment, which allow us to compare the anti-AChE potencies among different plant extracts and/or isolated compounds (Colovic et al., 2013).

A total of 207 original studies were retrieved, from which 71 were considered appropriate. All the species Latin names were validated at The Plant List (2013); version 1.1.; http://www.theplantlist.org/ (accessed 15th August, 2018). When the Latin name provided by the study diverged from that accepted at The Plant List, the species was identified by the accepted one followed by the former, which was reported as synonym, between parenthesis. To improve the readability of the text, the identity of the plant taxonomist(s) for each species is informed only in Table 1, excepting those mentioned as the source of isolated compounds, but whose extracts were not assayed.

**Table 1:** Plant extracts with *in vitro* anticholinesterase activity assessed by Ellman's Method reported in Pubmed from 2007 to 2018 (1st semester).

**Plant species (Families)**	**Type of extract or fraction (plant's part)**	**IC_50_ (μg/mL)**	**Toxicological assessment^#^**	**References**
*Scadoxus puniceus* (L.) Friis & Nordal (Amaryllidaceae)	Ethyl acetate extract (bulb)	0.3	Not assessed	Adewusi and Steenkamp, 2011
*Lannea schweinfurthii* Engl. (Anacardiaceae)	Ethyl acetate extract (root)	0.3	Not assessed	Adewusi and Steenkamp, 2011
*Carpolobia lutea* G. Don (Polygalaceae)	Ethyl acetate fraction (root)	0.3	≤ 100 μg/mL	Nwidu et al., 2017
*Xysmalobium undulatum* (L.) W. T. Aiton (Apocynaceae)	Ethyl acetate extract (root)	0.5	Not assessed	Adewusi and Steenkamp, 2011
*Phlegmariurus tetragonus* (Hook. & Grev.) B. Øllg. (Lycopodiaceae)^*^	Alkaloidal fraction (aerial parts)	0.9	Not assessed	Armijos et al., 2016
*Esenbeckia leiocarpa* Engl. (Rutaceae)	Alkaloidal fraction (stems)	1.6	Not assessed	Cardoso-Lopes et al., 2010
*Melissa officinalis* L. (Lamiaceae)	Ethanolic extract (leaves)	1.7	Not assessed	Dastmalchi et al., 2009
*Carpolobia lutea* G. Don (Polygalaceae)	Aqueous fraction (root)	2	≤ 100 μg/mL	Nwidu et al., 2017
*Crinum bulbispermum* (Burm. f.) Milne-Redh. & Schweick. (Amaryllidaceae)	Ethyl acetate extract (bulb)	2.1	Not assessed	Adewusi and Steenkamp, 2011
*Morus alba* L. (Moraceae)	Ethyl acetate fraction (root-bark)	2.5	Not assessed	Kuk et al., 2017
*Angelica decursiva* (Miq.) Franch. & Sav. (Apiaceae)	Aqueous fraction (whole plant)	2.6	Not assessed	Ali et al., 2015
*Carpolobia lutea* G. Don (Polygalaceae)	Methanolic extract (root)	3	≤ 100 μg/mL	Nwidu et al., 2017
*Buchanania axillaris* (Desr.) Ramamoorthy (Anacardiaceae)	Methanolic extract (aerial parts)	4.9	Not assessed	Penumala et al., 2018
*Salvia miltiorrhiza* Bunge (Lamiaceae)	Ethanolic extract (whole plant)	5.0	Not assessed	Lin et al., 2008
*Huperzia serrata* (Thunb.) Trevis. (Lycopodiaceae)	Alkaloids fraction (whole plant)	6.0	Not assessed	Ohba et al., 2015
*Esenbeckia leiocarpa* Engl. (Rutaceae)	Hexanic fraction (stems)	6.0	Not assessed	Cardoso-Lopes et al., 2010
*Angelica decursiva* (Miq.) Franch. & Sav. (Apiaceae)	Buthanolic fraction (whole plant)	6.0	Not assessed	Ali et al., 2015
*Berberis aetnensis* C. Presl (Berberidaceae)	Methanolic fraction (root)	7.6	Not assessed	Bonesi et al., 2013
*Senna obtusifolia (L.) H. S. Irwin & Barneby*. (Leguminosae)	Ethyl acetate fraction (leaves)	9.4	Not assessed	Jung et al., 2016
*Angelica decursiva* (Miq.) Franch. & Sav. (Apiaceae)	Ethyl acetate fraction (whole plant)	9.7	Not assessed	Ali et al., 2015
*Senna obtusifolia (L.) H.S. Irwin & Barneby*. (Leguminosae)	Buthanolic fraction (leaves)	9.9	Not assessed	Jung et al., 2016
*Zanthoxylum davyi* Waterm. (Rutaceae)	Methanolic extract (roots)	10	Not assessed	Adewusi and Steenkamp, 2011
*Ziziphus mucronata* Willd. (Rhamnaceae)	Ethyl acetate extract (root)	11.2	Not assessed	Adewusi and Steenkamp, 2011
*Morus alba* L. (Moraceae)	Methanolic extract (root-bark)	11.4	Not assessed	Kuk et al., 2017
*Zanthoxylum davyi* Waterm. (Rutaceae)	Ethyl acetate extract (roots)	11.6	Not assessed	Adewusi and Steenkamp, 2011
*Buchanania axillaris* (Desr.) Ramamoorthy (Anacardiaceae)	Chloroform fraction (aerial parts)	12.3	Not assessed	Penumala et al., 2018
*Senna obtusifolia (L.) H.S. Irwin & Barneby*. (Leguminosae)	Chloroform fraction (leaves)	12.7	Not assessed	Jung et al., 2016
*Morus alba* L. (Moraceae)	Chloroform fraction (root-bark)	13.4	Not assessed	Kuk et al., 2017
*Angelica decursiva* (Miq.) Franch. & Sav. (Apiaceae)	Chloroform fraction (whole plant)	13.7	Not assessed	Ali et al., 2015
*Senna obtusifolia (L.) H. S. Irwin & Barneby*. (Leguminosae)	Aqueous fraction (leaves)	14.5	Not assessed	Jung et al., 2016
*Crinum bulbispermum* (Burm. f.) Milne-Redh. & Schweick. (Amaryllidaceae)	Methanolic extract (bulb)	14.8	Not assessed	Adewusi and Steenkamp, 2011
*Scabiosa arenaria* Forssk. (Caprifoliaceae)	Ethyl acetate fraction (stem and leaves)	16	Not assessed	Besbes Hlila et al., 2013
*Angelica decursiva* (Miq.) Franch. & Sav. (Apiaceae)	Methanolic extract (whole plant)	16.6	Not assessed	Ali et al., 2015
*Berberis libanotica* Ehrenb. ex C.K. Schneid. (Berberidaceae)	Methanolic fraction (root)	16.9	Not assessed	Bonesi et al., 2013
*Pavetta indica* L. (Rubiaceae)	Methanolic extract (aerial parts)	17.8	Not assessed	Penumala et al., 2017
*Zephyranthes carinata* Herb. (Amaryllidaceae)	Alkaloidal fraction (bulb)	18.0	Not assessed	Cortes et al., 2015
*Crinum jagus (J. Thomps.)* Dandy (Amaryllidaceae)	Alkaloidal fraction (bulb)	18.3	≤ 28.7 μg/mL	Cortes et al., 2015
*Adenia gummifera* (Harv.) Harms (Passifloraceae)	Ethyl acetate extract (root)	18.9	Not assessed	Adewusi and Steenkamp, 2011
*Berberis libanotica* Ehrenb. ex C.K. Schneid. (Berberidaceae)	Methanolic extract (root)	21.7	Not assessed	Bonesi et al., 2013
*Huperzia squarrosa* (G. Forst.) Trevis. (Lycopodiaceae)	Ethyl acetate fraction (aerial parts)	23.4	Not assessed	Tung et al., 2017
*Berberis aetnensis* C. Presl (Berberidaceae)	Alkaloidal fraction (root)	24.5	Not assessed	Bonesi et al., 2013
*Ochna obtusata* DC. (Ochnaceae)	Chloroform fraction (aerial parts)	25.7	Not assessed	Penumala et al., 2017
*Gossypium herbaceum* L. (Malvaceae)	Hydroalcoholic extracts (flowers)	28.1	Not assessed	Zhao et al., 2013
*Hippeastrum puniceum* (Lam.) Voss. (Amaryllidaceae)	Alkaloid fraction (bulb)	28.1	≤ 28.7 μg/mL	Cortes et al., 2015
*Hemidesmus indicus* (L.) R. Br. ex Schult. (Apocynaceae)	Chloroform fraction (aerial part)	28.1	Not assessed	Penumala et al., 2018
*Scabiosa arenaria* Forssk. (Caprifoliaceae)	Buthanolic fraction (stems and leaves)	29.0	Not assessed	Besbes Hlila et al., 2013
*Senna obtusifolia (L.) H.S. Irwin & Barneby*. (Leguminosae)	Methanolic fraction (leaves)	29.2	Not assessed	Jung et al., 2016
*Morus alba* L. (Moraceae)	Buthanolic fraction (root-bark)	36.6	Not assessed	Kuk et al., 2017
*Ficus sur Forssk*. (Moraceae)	Ethyl acetate extract (fruit)	31.9	Not assessed	Adewusi and Steenkamp, 2011
*Rumex hastatus* D. Don (Polygonaceae)	Essential Oils (aerial parts)	32.5	Not assessed	Ahmad et al., 2016
*Acalypha alnifolia* Klein ex Willd. (Euphorbiaceae)	Chloroform fraction (aerial parts)	32.9	Not assessed	Penumala et al., 2017
*Olax nana* Wall. ex Benth. (Olacaceae)	Methanolic extract (leaves)	33.2	Not assessed	Ovais et al., 2018
*Nelumbo nucifera* Gaertn. (Nelumbonaceae)	Buthanolic fraction (leaves)	33.2	Not assessed	Jung et al., 2015
*Persicaria hydropiper* (L.) Delarbre. (Polygonaceae)^**^	Hexanic fraction (whole plant)	35.0	Not assessed	Ayaz et al., 2014
*Berberis aetnensis* C. Presl (Berberidaceae)	Hexanic fraction (root)	36.5	Not assessed	Bonesi et al., 2013
*Crinum bulbispermum* (Burm. f.) Milne-Redh. & Schweick. (Amaryllidaceae)	Ethyl acetate extract (root)	39.3	Not assessed	Adewusi and Steenkamp, 2011
*Huperzia brevifolia* (Grev. & Hook.) Holub (Lycopodiaceae)	Alkaloidal fraction (aerial parts)	39.6	Not assessed	Armijos et al., 2016
*Piper capense* L. f. (Piperaceae)	Ethyl acetate extract (root)	40.7	Not assessed	Adewusi and Steenkamp, 2011
*Searsia mysorensis* (G. Don) Moffett. (Anacardiaceae)	Chloroform fraction (aerial part)	41.3	Not assessed	Penumala et al., 2018
*Morus alba* L. (Moraceae)	Aqueous fraction (root-bark)	43.0	Not assessed	Kuk et al., 2017
*Hemidesmus indicus* (L.) R. Br. ex Schult. (Apocynaceae)	Methanolic extract (aerial part)	48.6	Not assessed	Penumala et al., 2018
*Huperzia squarrosa* (G. Forst.) Trevis. (Lycopodiaceae)	Buthanolic fraction (aerial parts)	50.1	Not assessed	Tung et al., 2017
*Esenbeckia leiocarpa* Engl. (Rutaceae)	Ethanolic extract (stems)	50.7	Not assessed	Cardoso-Lopes et al., 2010
*Scabiosa arenaria* Forssk. (Caprifoliaceae)	Ethyl Acetate fraction (flowers)	51.0	Not assessed	Besbes Hlila et al., 2013
*Pavetta indica* L. (Rubiaceae)	Chloroform fraction (aerial parts)	52.1	Not assessed	Penumala et al., 2017
*Polygonum hydropiper* L. (Polygonaceae)	Chloroform fraction (whole plant)	55.0	Not assessed	Ayaz et al., 2014
*Acalypha alnifolia* Klein ex Willd. (Euphorbiaceae)	Methanolic extrac (aerial parts)	59.2	Not assessed	Penumala et al., 2017
*Carpolobia lutea* G. Don (Polygalaceae)	Chloroform fraction (leaves)	60.0	≤ 100 μg/mL	Nwidu et al., 2017
*Pavetta indica* L. (Rubiaceae)	Buthanolic fraction (aerial parts)	60.1	Not assessed	Penumala et al., 2017
*Nelumbo nucifera* Gaertn. (Nelumbonaceae)	Ethylacetate fraction (leaves)	61.1	Not assessed	Jung et al., 2015
*Huperzia compacta* (Hook.) Trevis. (Lycopodiaceae)	Alkaloid fraction (aerial parts)	62.4	Not assessed	Armijos et al., 2016
*Acalypha alnifolia* Klein ex Willd. (Euphorbiaceae)	Aqueous fraction (aerial parts)	64.8	Not assessed	Penumala et al., 2017
*Nelumbo nucifera* Gaertn. (Nelumbonaceae)	Chloroform fraction (leaves)	67.3	Not assessed	Jung et al., 2015
*Buchanania axillaris* (Desr.) Ramamoorthy (Anacardiaceae)	Buthanolic fraction (aerial parts)	67.5	Not assessed	Penumala et al., 2018
*Scabiosa arenaria* Forssk. (Caprifoliaceae)	Buthanolic fraction (flowers)	74.0	Not assessed	Besbes Hlila et al., 2013
*Rumex hastatus* D. Don (Polygonaceae)	Chloroform fraction (whole plant)	75.0	Not assessed	Ahmad et al., 2015
*Scabiosa arenaria* Forssk. (Caprifoliaceae)	Methanolic extract (flowers)	80.0	Not assessed	Besbes Hlila et al., 2013
*Carpolobia lutea* G. Don (Polygalaceae)	Ethanolic fraction (leaves)	81.0	≤ 100 μg/mL	Nwidu et al., 2017
*Ochna obtusata* DC. (Ochnaceae)	Methanolic extrac (aerial parts)	82.2	Not assessed	Penumala et al., 2017
*Berberis libanotica* Ehrenb. ex C.K. Schneid. (Berberidaceae)	Alkaloidal extract (root)	82.4	Not assessed	Bonesi et al., 2013
*Searsia mysorensis* (G. Don) Moffett. (Anacardiaceae)	Buthanolic fraction (aerial part)	83.5	Not assessed	Penumala et al., 2018
*Searsia mysorensis* (G. Don) Moffett. (Anacardiaceae)	Aqueous fraction (aerial part)	93.7	Not assessed	Penumala et al., 2018
*Berberis libanotica* Ehrenb. ex C.K. Schneid. (Berberidaceae)	Hexanic extract (root)	95.5	Not assessed	Bonesi et al., 2013
*Jatropha gossypifolia* L. (Euphorbiaceae)	Ethyl acetate fraction (leaves)	95.7	Not assessed	Saleem et al., 2016
*Polygonum hydropiper* L. (Polygonaceae)	Aqueous fraction (whole plant)	100.0	Not assessed	Ayaz et al., 2014
*Pavetta indica* L. (Rubiaceae)	Aqueous fraction (aerial parts)	100.4	Not assessed	Penumala et al., 2017
*Stemona sessilifolia* (Miq.) Miq. (Stemonaceae)	Alkaloidal extracts (root)	102.6	Not assessed	Lai et al., 2013
*Huperzia squarrosa* (G. Forst.) Trevis. (Lycopodiaceae)	Ethanolic extract (aerial parts)	112.2	Not assessed	Tung et al., 2017
*Hemidesmus indicus*(L.) R. Br. ex Schult. (Apocynaceae)	Buthaolic fraction (aerial part)	113.5	Not assessed	Penumala et al., 2018
*Rumex hastatus* D. Don (Polygonaceae)	Ethyl acetate fraction (whole plant)	115.0	Not assessed	Ahmad et al., 2015
*Nelumbo nucifera* Gaertn. (Nelumbonaceae)	Aqueous fraction (leaves)	119.6	Not assessed	Jung et al., 2015
*Persicaria hydropiper* (L.) Delarbre. (Polygonaceae)^***^	Essential Oils (leaves)	130.0	Not assessed	Ayaz et al., 2015
*Hemidesmus indicus*(L.) R. Br. ex Schult. (Apocynaceae)	Aqueous fraction (aerial part)	129.4	Not assessed	Penumala et al., 2018
*Buchanania axillaris* (Desr.) Ramamoorthy (Anacardiaceae)	Aqueous fraction (aerial parts)	136.2	Not assessed	Penumala et al., 2018
*Carpolobia lutea* G. Don (Polygalaceae)	Ethanolic extract (stem-bark)	140.0	≤ 100 μg/mL	Nwidu et al., 2017
*Carpolobia lutea* G. Don (Polygalaceae)	Hexanic fraction oil (stem)	140.0	≤ 100 μg/mL	Nwidu et al., 2017
*Carpolobia lutea* G. Don (Polygalaceae)	Methanolic fraction (stem)	142.0	≤ 100 μg/mL	Nwidu et al., 2017
*Elatostema papillosum* Wedd. (Urticaceae)	Methanolic extract (leaves)	165.4	Not assessed	Reza et al., 2018
*Scabiosa arenaria* Forssk. (Caprifoliaceae)	Aqueous fraction (flowers)	170	Not assessed	Besbes Hlila et al., 2013
*Ochna obtusata* DC. (Ochnaceae)	Buthanolic fraction (aerial parts)	174.4	Not assessed	Penumala et al., 2017
*Jatropha gossypifolia* L. (Euphorbiaceae)	Dichloromethane extract (root)	176.0	Not assessed	Saleem et al., 2016
*Nelumbo nucifera* Gaertn. (Nelumbonaceae)	Methanolic fraction (leaves)	184.5	Not assessed	Jung et al., 2015
*Rumex hastatus* D. Don (Polygonaceae)	Methanolic extract (whole plant)	218.0	Not assessed	Ahmad et al., 2015
*Jatropha gossypifolia* L. (Euphorbiaceae)	Methanolic extract (root)	222.0	Not assessed	Saleem et al., 2016
*Polygonum hydropiper* L. (Polygonaceae)	Essential Oils (flowers)	225.0	Not assessed	Ayaz et al., 2015
*Scabiosa arenaria* Forssk. (Caprifoliaceae)	Methanolic extract (stems and leaves)	230.0	Not assessed	Besbes Hlila et al., 2013
*Diplotaxis simplex* Asch. ex Rohlfs (Brassicaceae)	Aqueous extract (seeds)	233.0	Not assessed	Bahloul et al., 2016
*Persicaria minor* (Huds.) Opiz. (Polygonaceae)^****^	Aqueous extract (leaves)	234.0	Not assessed	Ahmad et al., 2014
*Scabiosa arenaria* Forssk. (Caprifoliaceae)	Buthanolic fraction (fruits)	240.0	Not assessed	Besbes Hlila et al., 2013
*Polygonum hydropiper* L. (Polygonaceae)	Buthanolic fraction (whole plant)	240.0	Not assessed	Ayaz et al., 2014
*Huperzia squarrosa* (G. Forst.) Trevis. (Lycopodiaceae)	Hexanic fraction (aerial parts)	257.0	Not assessed	Tung et al., 2017
*Acalypha alnifolia* Klein ex Willd. (Euphorbiaceae)	Buthanolic fraction (aerial parts)	257.5	Not assessed	Penumala et al., 2017
*Atriplex laciniata* L. (Amaranthaceae)	Aqueous fraction (whole plant)	267.0	Not assessed	Kamal et al., 2015
*Scabiosa arenaria* Forssk. (Caprifoliaceae)	Methanolic extract (fruits)	270.0	Not assessed	Besbes Hlila et al., 2013
*Atriplex laciniata* L. (Amaranthaceae)	Ethyl acetate fraction (whole plant)	270.0	Not assessed	Kamal et al., 2015
*Atriplex laciniata* L. (Amaranthaceae)	Methanolic extract (whole plant)	280.0	Not assessed	Kamal et al., 2015
*Jatropha gossypifolia* L. (Euphorbiaceae)	Dichloromethane extract (leaves)	289.0	Not assessed	Saleem et al., 2016
*Justicia adhatoda L*. (Acanthaceae)	Methanolic extract (leaves)	294.0	Not assessed	Ali et al., 2013
*Polygonum hydropiper* L. (Polygonaceae)	Ethyl acetate fraction (whole plant)	310.0	Not assessed	Ayaz et al., 2014
*Atriplex laciniata* L. (Amaranthaceae)	Hexanic fraction (whole plant)	310.0	Not assessed	Kamal et al., 2015
*Diplotaxis harra* (Forssk.) Boiss. (Brassicaceae)	Aqueous extract (seeds)	313.0	Not assessed	Bahloul et al., 2016
*Polygonum hydropiper* L. (Polygonaceae)	Methanolic extract (whole plant)	330.0	Not assessed	Ayaz et al., 2014
*Polygonum minus Huds. (Polygonaceae)*	Methanolic extract (leaves)	342.8	Not assessed	Ahmad et al., 2014
*Ochna obtusata* DC. (Ochnaceae)	Aqueous fraction (aerial parts)	369.1	Not assessed	Penumala et al., 2017
*Atriplex laciniata* L. (Amaranthaceae)	Chloroform fraction (whole plant)	390.0	Not assessed	Kamal et al., 2015
*Scabiosa arenaria* Forssk. (Caprifoliaceae)	Aqueous fraction (stems and leaves)	410.0	Not assessed	Besbes Hlila et al., 2013
*Diplotaxis simplex* Asch. ex Rohlfs (Brassicaceae)	Aqueous extract (flowers)	420.0	Not assessed	Bahloul et al., 2016
*Salsola vermiculata* L. (Amaranthaceae)	Methanol extract (roots)	450.0	Not assessed	Rasheed et al., 2013
*Polygonum minus* Huds. *(Polygonaceae)*	Dichloromethane extract (leaves)	478.0	Not assessed	Ahmad et al., 2014
*Scabiosa arenaria* Forssk. (Caprifoliaceae)	Ethyl acetate fraction (fruits)	500.0	Not assessed	Besbes Hlila et al., 2013
*Polygonum minus* Huds. (Polygonaceae)	Aqueous extract (stem)	581.0	Not assessed	Ahmad et al., 2014
*Salvia leriifolia* Benth. (Lamiaceae)	Hexane fraction (leaves)	590.0	Not assessed	Loizzo et al., 2010
*Jacaranda caroba* (Vell.) DC. (Bignoniaceae)	Aqueous extract (leaves)	670.2	Not assessed	Ferreres et al., 2013
*Polygonum minus* Huds. *(Polygonaceae)*	Dichloromethane extract (leaves)	770.0	Not assessed	Ahmad et al., 2014
*Diplotaxis harra* (Forssk.) Boiss. (Brassicaceae)	Aqueous extract (flowers)	760.0	Not assessed	Bahloul et al., 2016
*Polygonum minus Huds. (Polygonaceae)*	Methanolic extract (stem)	809.0	Not assessed	Ahmad et al., 2014
*Salvia leriifolia* Benth. (Lamiaceae)	Dichloromethane fraction (leaves)	840.0	Not assessed	Loizzo et al., 2010
*Salvia leriifolia* Benth. (Lamiaceae)	Ehtyl acetate fraction (leaves)	870.0	Not assessed	Loizzo et al., 2010
*Rhizophora lamarckii* Montrouz. (Rhizophoraceae)	Methanol extract (leaves)	910.0	Not assessed	Suganthy et al., 2009
*Polygonum minus* Huds*. (Polygonaceae)*	Ethanolic extract (leaves)	910.0	Not assessed	Ahmad et al., 2014
*Polygonum minus* Huds. *(Polygonaceae)*	Ethanolic extract (stem)	930.0	Not assessed	Ahmad et al., 2014
*Jacaranda caroba* (Vell.) DC. (Bignoniaceae)	Hydromethanolic extracts (leaves)	1000.4	Not assessed	Ferreres et al., 2013

*AChE, acetylcholinesterase; IC_50_, half maximal inhibition concentration*.

* *Syn, Huperzia tetragona (Hook. & Grev.) Trevis*.

** *Syn, Polygonum hydropiper L*.

*** *Syn, Polygonum hydropiper L*.

**** *Syn, Polygonum minus Huds*.

# *Maximal concentration assessed for the absence of cytotoxicity*.

*Plant species and their respective extracts were classified in accordance with the criteria described at Section Plant Species With Anti-Acetylcholinesterase Activity in: high potency (green background), moderate potency (orange background), and low potency (red background)*.

## Plant species with anti-acetylcholinesterase activity

Amaryllidaceae is the leading family of genera holding anti-AChE activity, particularly *Galanthus* spp., which are the primordial source of galantamine (Heinrich, 2010). However, subsequently to galantamine's approval for the treatment of mild-to-moderate AD in 2001, a plethora of species have been assessed in a pursuit of new AChEi. In our survey timeframe, a total of 39 studies reporting the anti-AChE activity for 51 species, from 29 different families, were considered. The most prevalent families were Amaryllidaceae, Lycopodiaceae, and Polygonaceae, contributing with 5, 5, and 4 species, respectively. Noteworthy, *Huperzia* spp. keep drawing ethnopharmacology researchers' attention, despite the consistent basic and clinical evidence already available for Huperzine A on AD treatment (Ha et al., 2011; Sahoo et al., 2018).

Table 1 summarizes the contemplated species, which were classified in three categories, in accordance to the IC_50_ values determined for their respective extracts/fractions: high potency, IC_50_ < 20 μg/mL; moderate potency, 20 < IC_50_ < 200 μg/mL; and low potency, 200 < IC_50_ < 1,000 μg/mL. Those cutoffs were set according to the average IC_50_ value described for galantamine in the literature (~ 2 μM or 0.575 μg/mL) multiplied by a factor of 10 (Lopez et al., 2002; Ingkaninan et al., 2003; Berkov et al., 2008). Similar criteria have been previously applied by Murray et al. (2013), excepting that they included studies reporting only the maximal anti-AChE inhibitory activity and set the cutoff for low potency at IC_50_ > 500 μg/mL.

Twenty-four plant species fell into high potency category, with IC_50_ values varying from 0.3 μg/mL for ethyl acetate bulb extract of *Scadoxus puniceus* (Amaryllidaceae), ethyl acetate root extract of *Lannea schweinfurthii* (Anacardiaceae; Adewusi and Steenkamp, 2011), and ethyl acetate root fraction of *Carpolobia lutea* G. Don (Polygalaceae; Nwidu et al., 2017); to 18.9 μg/mL for the ethyl acetate root extract of *Adenia gummifera (Harv.) Harms (Passifloraceae;* Adewusi and Steenkamp, 2011; Table 1). Both *S. puniceus* and *L. schweinfurthii* ethyl acetate extracts showed very-limited antioxidant activity, leading the authors to attribute the strong anti-AChE activity to the extract alkaloid-content (Adewusi and Steenkamp, 2011). However, primary extraction with ethyl acetate hardly renders alkaloid-rich extracts, which demands an extraction scheme outlined to adjustable acid and basic pH values during partitioning (Sarker et al., 2005), therefore further validation for those species is advisable. Notwithstanding, analyzing the solvents employed for preparation of the potent extracts within this category (Table 1), there is no direct correlation between solvent polarity and anti-AChE activity, supporting the assumption that non-alkaloidal secondary metabolites, such as terpenoids, flavonoids and other phenolic compounds, would be as active as the classic alkaloidal AChEi (Murray et al., 2013).

For instance, the ethyl acetate root fraction of *Carpolobia lutea* (Polygalaceae)–whose total phenolic content was 296.5 mg EAG/g–presented IC_50_ = 0.3 μg/mL (Nwidu et al., 2017); virtually the same value determined to the essential oil from *Salvia leriifolia* (Lamiaceae) aerial parts, which presented IC_50_ = 0.32 μL/mL and had camphor (10.5%), 1,8-cineole (8.6%), camphene (6.2%) and α-pinene (4.7%) as main components (Loizzo et al., 2009). Contrarily, the n-Hexane whole plant fraction of *Polygonum hydropiper* (Polygonaceae) crude extract presented moderate anti-AChE activity with IC_50_ = 35 μg/mL (Ayaz et al., 2014), meanwhile the essential oil from its leaves showed a potency nearly four times lower (IC_50_ = 120 μg/mL; Ayaz et al., 2015). The alkaloid fraction of *Esenbeckia leiocarpa* (Rutaceae), obtained by acid-base partition of the ethanol stem extract, presented IC_50_ = 1.6 μg/mL, which corresponded to an inhibitory potency 30-fold higher than the original crude extract (IC_50_ = 50.7 μg/mL; Cardoso-Lopes et al., 2010). Still, the assessment of anti-AChE activity of *Berberis aetnensis* and *Berberis libanotica* root extracts, whose major constituent was the alkaloid berberine, showed 3-fold higher activity for the methanol fraction (IC_50_ = 7.6 and 16.9 μg/mL, respectively) than for alkaloid-rich fraction (IC_50_ = 24.5 and 82.4 μg/mL, respectively), supporting the synergy between alkaloid and non-alkaloid components within methanol fraction from both species (Bonesi et al., 2013).

*Huperzia* spp. (Lycopodiaceae) have been used for over 1,000 years in China for diverse neuronal- and cognitive-based illnesses (Ma et al., 2007), becoming of major interest for the pharmaceutical industry upon the isolation of the alkaloid Huperzine A from *H. serrata* (Liu et al., 1986). Thenceforth, huge research has focused on the isolation of Huperzine A and other Lycopodium alkaloids from *Huperzia* spp. and other Lycopodiaceae species (Ha et al., 2011; Damar et al., 2016; Sahoo et al., 2018). In spite of that, our survey retrieved recent relevant studies on anti-AChE activity of five *Huperzia* spp.: *H. serrata* (Ohba et al., 2015), *H. squarrosa* (Tung et al., 2017), *H. brevifolia, H. compacta*, and *H. tetragona* (Armijos et al., 2016; Table 1). In the study by Ohba et al. (2015), alkaloid enriched fraction of *H. serrata* aerial parts, whose major alkaloidal constituent was Huperzine A (~0.5%), presented IC_50_ = 5.96 μg/mL. On the other hand, in the study by Armijos et al. (2016), alkaloid fraction of *H. tetragona* aerial parts strongly inhibited AChE (IC_50_ = 0.9 μg/mL), meanwhile *H. brevifolia* and *H. compacta* presented moderate potency (IC_50_ = 39.6 and 62.4 μg/mL, respectively). The authors ascribed the high potency of *H. tetragona* to other Lycopodium alkaloids, mainly lycopodine, 6-OH-lycopodine and des-*N*-methyl-α-obscurine, since Huperzine A was not detected in any of the assessed species. Tung et al. (2017) assessed anti-AChE activity in three different fractions obtained from the ethanol extract of *H. squarrosa* aerial parts. EtOAc and BuOH fractions presented moderated activity, whose IC_50_ values were 23.44 and 50.11 μg/mL, respectively. The n-hexane fraction otherwise presented the lowest AChE inhibitory activity (IC_50_ = 257.03 μg/mL).

As showed in the abovementioned studies, anti-AChE activity of plant extracts is significantly variable regardless of the predominant secondary metabolite class or the polarity of the extracting solvent. To cope with these limitations and still screen potentially applicable species, most researchers have also assessed the extracts antioxidant capacity, in order to demonstrate their dual efficacy. Although the present mini-review does not aim to discuss antioxidant aspects, it is noticeable that most studies cited in Table 1 have either quantified total phenolic content or measured antioxidant capacity in their extracts. Such assessments require appropriate methods that address the mechanism of antioxidant activity and focus on the kinetics of the reactions involving the antioxidants (Amorati and Valgimigli, 2018). Contrariwise, phenolic content was predominantly measured by Folin-Ciocalteu method, which also quantifies nonphenolic compounds, such as aromatic amino acids, sugars, ascorbic acid, and organic acids (Pueyo and Calvo, 2009), reason why it is not advisable for total phenol quantification. Similarly, antioxidant capacity was mostly assessed by trapping of the radicals DPPH^•^ and ABTS^•+^, which are non-biologically relevant oxidants (Amorati and Valgimigli, 2018). Thus, most plant extracts propelled as dually efficient (Anti-AChE and antioxidant) much probably deserve a biological approach to characterize their preventive instead of scavengering antioxidant capacity.

## Natural AChEi compounds

**Figure 1:**
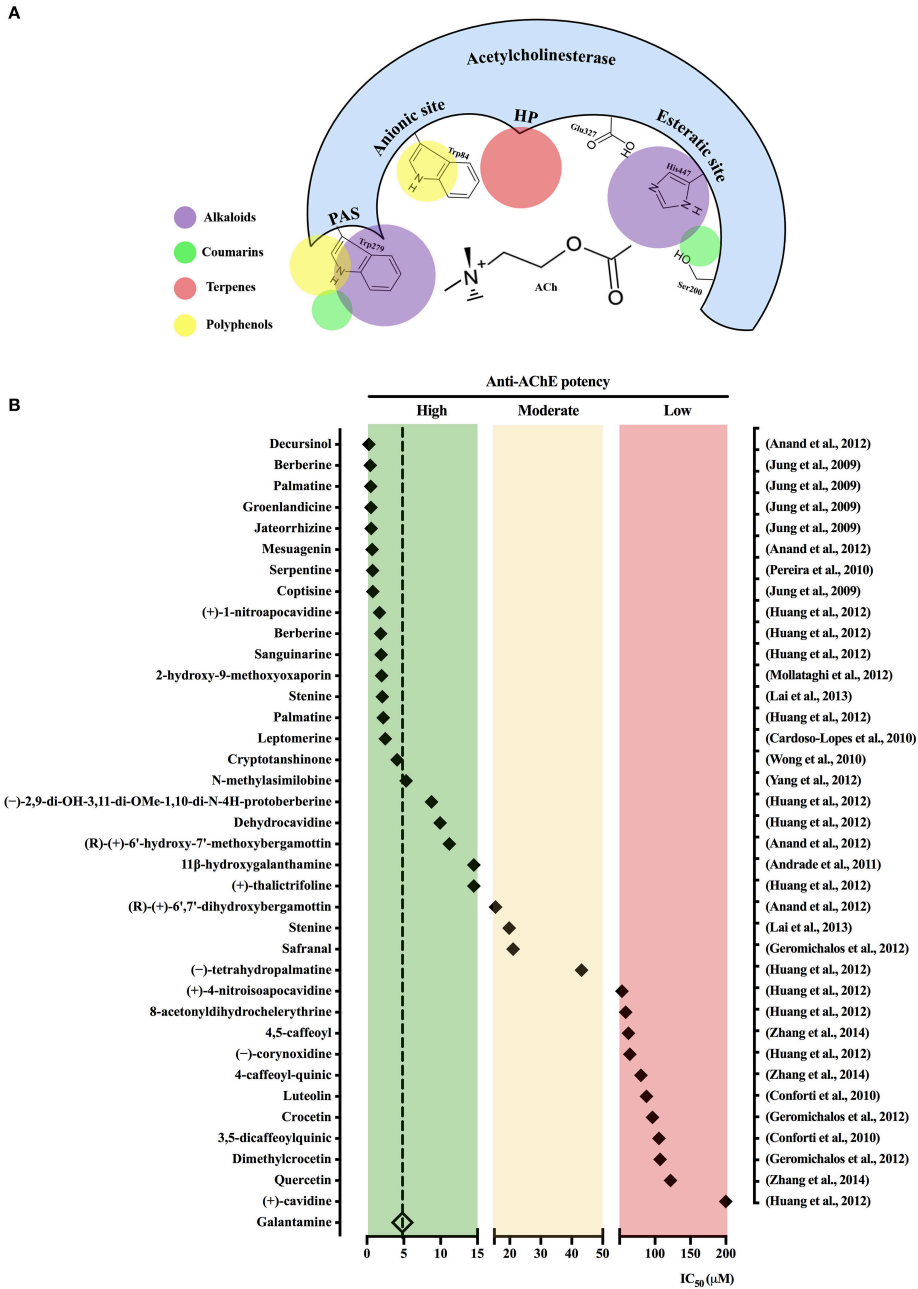
Schematic view of acetylcholinesterase active binding sites for the main natural acetylcholinesterase inhibitors (AChEi) classes. **(A)** Acetylcholinesterase gorge pocket composed by the following binding sites: esteratic site, anionic site, peripheral anionic site (PAS), as well as hydrophobic pocket (HP) is shown. Colored circles represent the main binding sites for compounds pertaining to the indicated classes. **(B)** Natural AChEi reported in Pubmed from 2007 to 2018 (1st semester) were classified in accordance with the criteria described at Section Natural AChEi Compounds in: high potency (green background), moderate potency (orange background), and low potency (red background). The diamond symbol represents the IC_50_ value for each compound (in the left) as described in the respective study (in the right).

The active site of AChE contains two main subsites, the “esteratic” and “anionic” subsites, corresponding to the catalytic machinery and the choline-binding pocket, respectively. As illustrated in Figure 1A, the “esteratic” subsite consists in a histidine residue (His_447_), whereas the “anionic” subsite is an tryptophan residue (Trp_84_) able to bind quaternary ligands, which may act as competitive inhibitors (Dvir et al., 2010). Most of natural AChEi reported during our delimited survey period belong to the alkaloid group. Anti-AChE activity of alkaloids is ascribed to their complex nitrogen structures, which once positively charged bind to the “anionic” subsite on AChE active site (Hostettmann et al., 2006; Houghton et al., 2006). For instance, galantamine inhibits AChE by stably binding to Trp_84_, as well as phenylalanine residues on the acyl-binding pocket (Greenblatt et al., 1999). On the other hand, non-alkaloidal AChEi, which include terpenes, flavonoids and other phenolic compounds, seem to act as non-competitive inhibitors that bind to peripheral anionic sites (PAS) mainly represented by the residues Tyr_70_, Asp_74_, Try_121_, Trp_279_, and Tyr_334_ (Johnson and Moore, 2006).

Figure 1B shows the isolated compounds identified as potential natural AChEi, which were classified in three categories, in accordance to their IC_50_ values: high potency, IC_50_ < 15 μM; moderate potency, 15 < IC_50_ < 50 μM; and low potency, 50 < IC_50_ < 1,000 μM. As a comparative reasoning, IC_50_ values described for galantamine in the surveyed studies where it was used as positive control were averaged, resulting in IC_50_ = 4.82 ± 1.29 μM. Studies describing discrepant IC_50_ values for galantamine were not considered. Sixteen compounds presented anti-AChE potency higher than galantamine, which include 01 terpene, 2 coumarins, and 13 alkaloids. Other 20 compounds, additionally pertaining to flavonoids and phenolic acids, were also selected with IC_50_ ranging from 5.33 to >200 μM (Figure 1B). The dihydropyranocoumarin decursinol isolated from *Angelica gigas* Nakai (Apiaceae) was the most potent AChEi (IC_50_ = 0.28 μM; Anand et al., 2012), such high potency had been previously attributed to characteristics of cyclization of the isoprenyl unit at C-6 and the functional groups attached to the coumarin nucleus, which differ from other coumarins (Kang et al., 2001).

The major alkaloids with recognized anti-AChE activity are the classical galantamine and huperzine A, which have been elegantly reviewed by Gulcan et al. (2015) and Qian and Ke (2014), respectively. However, other recently described alkaloids of various subclasses deserve special emphasis because of their important inhibitory action on AChE. Jung et al. (2009) assessed the anti-AChE activity of five protoberberine alkaloids isolated from the rhizome of *Coptis* spp. (berberine, palmatine, groenlandicine, jateorrhizine, and coptisine), with IC_50_ ranging from 0.44 to 0.80 μM. Interestingly, groenlandicine also strongly inhibited the enzyme responsible for cleaving the β-site of the amyloid precursor protein, adding an important property against AD pathogenesis (Jung et al., 2009). The potentialities of protoberberines alkaloids as natural AChEi were further supported by isolation of 12 isoquinoline alkaloids, including two new nitrotetrahydroprotoberberines (2,9-dihydroxy-3,11-dimethoxy-1,10-dinitrotetrahydroprotoberberine and 4-nitroisoapocavidine), from *Corydalis saxicola* Bunting (Papaveraceae). All the alkaloids were selectively active against AChE with IC_50_ < 10 μM. Structure-activity relationship study indicated that potency differences were related to the presence of phenolic hydroxy groups, which could reduce the anti-AChE activity, whereas nitro substitutions at ring A, especially at C-1, in the tetrahedroprotoberberines could increase it (Huang et al., 2012).

Studies on the molecular mechanisms by which natural AChEi interact with AChE binding subsites are still scant. Nevertheless, some studies have offered important insights on this matter. Serpentine, the main alkaloid found in the roots of *Catharanthus roseus* (L.) G. Don (Apocynaceae) presented high anti-AChE potency (IC_50_ = 0.77 μM), which was attributed to the binding of its quaternary nitrogen to an Asp residue at AChE peripheral anionic site (Pereira et al., 2010). Lai et al. (2013) when evaluating alkaloids from *Stemona sessilifolia* (Miq.) Miq. roots (Stemonaceae) identified the AChEi stenine B (IC_50_ = 2.10 μM) and stenine (IC_50_ = 19.8 μM). Authors attributed the stronger activity of stenine B to its ability to build hydrogen bonds with Tyr_130_, similarly to huperzine A. Lastly, bioactivity-guided chromatographic fractionation of *Nelumbo nucifera* Gaertn. (Nelumbonaceae) leaf extract led to isolation of three aporphine-type alkaloids, an important subclass of natural inhibitors of AChE. Amongst them, N-methylasimilobine displayed a significant anti-AChE activity with IC_50_ = 5.33 μM. According to their *in silico* studies, such potency was due to a hydroxyl group at the alkaloid C-2 position, which makes hydrogen bond with a carbonyl group on Ser_293_ in association with another hydrogen bond between its alkaloidal quaternary nitrogen and the hydroxyl group of Tyr_124_ (Yang et al., 2012).

*Salvia* spp. (Lamiaceae) have been used for centuries for its beneficial effects on memory disorders (Hamidpour et al., 2014). Wong et al. (2010) demonstrated that the diterpene cryptotanshinone extracted from the root of *Salvia miltiorrhiza* Bunge is a reversible inhibitor of human AChE (IC_50_ = 4.09 μM) and that chronic oral administration can reverse cognitive deficits induced by scopolamine in rats. Flavonoids, a heterogeneous group of polyphenols, are currently considered a prominent source of anti-AD compounds (Khan et al., 2018) because of their potential AChE inhibitory activity allied to the well-known antioxidant activity and low toxicity (Uriarte-Pueyo and Calvo, 2011). However, our survey did not identify any highly potent, and consequently prominent AChEi pertaining to the flavonoid class (Figure 1). For instance, luteolin and 3,5-dicaffeoylquinic acid, phenolic compounds extracted from *Phagnalon saxatile* Cass. (Compositae) exhibited low activity against AChE with an IC_50_ of 88.00 and 105 μM, respectively (Conforti et al., 2010).

## Clinical studies

Besides galantamine, huperzine A is the most clinically studied alkaloidal AChEi (Qian and Ke, 2014). The efficacy of huperzine A was demonstrated in the treatment of 447 patients with age-related memory impairment or dementia (Shu, 1998; Ma et al., 2007). However, in another phase II study, the results were not conclusive on its beneficial cognitive effects for patients with moderate AD, requiring further investigation (Rafii et al., 2011). A clinical trial with *Salvia officinalis* L. administered to patients with mild to moderate AD for a 16-weeks period led to improved cognitive performance (Perry et al., 2003). Of importance, *S. officinalis* also attenuated cognitive impairment in patients suffering from moderate to severe AD when used for up to 1 year. However, authors recognized that long-term efficacy, safety and administration strategy still require further investigation (Tune, 2001). *Salvia* spp. are particularly rich in terpenes, whose anti-AChE capacity has been assessed through enough pre-clinical tests, but are awaiting clinical trials (Rollinger et al., 2004; Kennedy and Scholey, 2006). On the other hand, a 22-weeks randomized, double-blind, multicenter trial, including 54 individuals suffering from mild-to-moderate AD, showed that daily intake of *Crocus sativus* L. (Iridaceae) dried extract (30 mg/day) significantly improved cognitive capacity comparable to that observed in donepezil-treated patients (Akhondzadeh et al., 2010).

## Toxicological studies

A recent systematic review and meta-analysis of 43 randomized placebo-controlled clinical trials showed that AChEi improved cognitive function, global symptomatology, and functional capacity, as well as decreased patients' mortality (Blanco-Silvente et al., 2017). However, patients taking AChEi presented higher discontinuation due to adverse events, denoting an important issue on anti-AChE therapy. As showed in Table 1, the majority of the plant extract-based studies mentioned in this mini-review has not assessed their toxicity in animals or humans, although species like *S. officinalis* (Kennedy and Scholey, 2006) and *P. hydropiper* (Huq et al., 2014) have been considered as non-toxic. Amongst the main natural AChEi compounds herein mentioned, berberine and safranal seem to ally more advantages than disadvantages. Nevertheless, berberine has been shown to cause mild gastrointestinal reactions, including diarrhea and constipation, besides other less frequent side effects (Imenshahidi and Hosseinzadeh, 2016); and safranal has toxic effects on hematological and biochemical indices, as well as induced embryonic malformation in animal's models at high doses (Bostan et al., 2017).

## Closing remarks and perspectives

The present mini-review demonstrated that during last decade several plant species and their potentially active compounds have been screened for anti-AChE activity. Amongst the most active extracts (Table 1), it is noticeable the use of extracting solvents of distinct polarities, which suggests that their active compounds might pertain to a wide range of secondary metabolites classes. However, having a look at the isolated substances summarized in Figure 1B, most high potent compounds assessed during this period pertain to alkaloid class, exception made to the highest potent decursinol, a dihydropyranocoumarin. Alkaloids indisputably are the most studied class of natural AChEi, what seemly has trapped the researcher's attention in this class when in pursuit of new potential AChEi candidates, a vision that urges to be changed. Notwithstanding, the search for secondary AD-relevant pharmacological properties, such as antioxidant, deserves experimental approaches addressing their capacity to prevent oxidants generation and oxidative damage, instead of their mere scavengering capacity.

Finally, despite the undoubted relevance of new AChEi discovery for AD palliative pharmacotherapy, there is scanty knowledge on their structure-activity relationships, as well as toxicological assessments that would enable them to phase II studies. For instance, berberine and related protoberberine alkaloids have been consistently assessed for their anti-AChE activity, but no phase II study has been conducted so far. Such knowledge is capital both to promote higher safety and to guide the design of new (semi-) synthetic AChEi. Thus, given the plethora of plant species and compounds already described, their assessment through clinical trials certainly represent the main barrier to be transposed in order to expand and improve the pharmacological care of AD patients.

## Author contributions

TS conceived the proposal, discussed mini-review's structure, surveyed and selected relevant articles, tabulated the data and drafted the manuscript. TG surveyed and selected relevant articles, tabulated the data. BP supervised articles selection, analysis and data tabulation. AC discussed mini-review's structure, supervised articles selection, analysis and data tabulation. AP conceived the proposal, discussed mini-review's structure, oriented the selection of relevant articles, analyzed tabulated data, and drafted the manuscript. All authors read and approved the final format of the manuscript.

### Conflict of interest statement

The authors declare that the research was conducted in the absence of any commercial or financial relationships that could be construed as a potential conflict of interest.

## References

[B1] Adewusi E. A. Steenkamp V. (2011). *In vitro* screening for acetylcholinesterase inhibition and antioxidant activity of medicinal plants from southern Africa. Asian Pacif. J. Trop. Med. 4, 829–835. 10.1016/S1995-7645(11)60203-4 22014742

[B2] Ahmad R. Baharum S. N. Bunawan H. Lee M. Mohd Noor N. Rohani E. R.. (2014). Volatile profiling of aromatic traditional medicinal plant, Polygonum minus in different tissues and its biological activities. Molecules 19, 19220–19242. 10.3390/molecules191119220 25420073 PMC6271663

[B3] Ahmad S. Ullah F. Ayaz M. Sadiq A. Imran M. (2015). Antioxidant and anticholinesterase investigations of Rumex hastatus D. Don: potential effectiveness in oxidative stress and neurological disorders. Biol. Res. 48:20 10.1186/s40659-015-0010-2 25857346 PMC4381421

[B4] Ahmad S. Ullah F. Sadiq A. Ayaz M. Imran M. Ali I. (2016). Chemical composition, antioxidant and anticholinesterase potentials of essential oil of Rumex hastatus D. Don collected from the North West of Pakistan. BMC Complement. Altern. Med. 16:29 10.1186/s12906-016-0998-z 26810212 PMC4727414

[B5] Akhondzadeh S. Shafiee Sabet M. Harirchian M. H. Togha M. Cheraghmakani H. Razeghi S.. (2010). A 22-week, multicenter, randomized, double-blind controlled trial of Crocus sativus in the treatment of mild-to-moderate Alzheimer's disease. Psychopharmacology 207, 637–643. 10.1007/s00213-009-1706-1 19838862

[B6] Ali M. Y. Jung H. A. Choi J. S. (2015). Anti-diabetic and anti-Alzheimer's disease activities of Angelica decursiva. Arch. Pharmacal. Res. 38, 2216–2227. 10.1007/s12272-015-0629-0 26152875

[B7] Ali S. K. Hamed A. R. Soltan M. M. Hegazy U. M. Elgorashi E. E. El-Garf I. A. (2013). *In-vitro* evaluation of selected Egyptian traditional herbal medicines for treatment of Alzheimer disease. BMC Complement. Alter. Med. 13:121 10.1186/1472-6882-13-121 PMC3701527 23721591

[B8] Amorati R. Valgimigli L. (2018). Methods to measure the antioxidant activity of phytochemicals and plant extracts. J. Agric. Food Chem. 66, 3324–3329. 10.1021/acs.jafc.8b01079 29557653

[B9] Anand P. Singh B. (2013). A review on cholinesterase inhibitors for Alzheimer's disease. Arch. Pharmacal Res. 36, 375–399. 10.1007/s12272-013-0036-3 23435942

[B10] Anand P. Singh B. Singh N. (2012). A review on coumarins as acetylcholinesterase inhibitors for Alzheimer's disease. Bioorg. Med. Chem. 20, 1175–1180. 10.1016/j.bmc.2011.12.042 22257528

[B11] Andrieu S. Coley N. Lovestone S. Aisen P. S. Vellas B. (2015). Prevention of sporadic Alzheimer's disease: lessons learned from clinical trials and future directions. Lancet Neurol. 14, 926–944. 10.1016/S1474-4422(15)00153-2 26213339

[B12] Armijos C. Gilardoni G. Amay L. Lozano A. Bracco F. Ramirez J.. (2016). Phytochemical and ethnomedicinal study of Huperzia species used in the traditional medicine of Saraguros in Southern Ecuador; AChE and MAO inhibitory activity. J. Ethnopharmacol. 193, 546–554. 10.1016/j.jep.2016.09.049 27686269

[B13] Ayaz M. Junaid M. Ahmed J. Ullah F. Sadiq A. Ahmad S. (2014). Phenolic contents, antioxidant and anticholinesterase potentials of crude extract, subsequent fractions and crude saponins from *Polygonum hydropiper* L. BMC Complement. Altern. Med. 14:145 10.1186/1472-6882-14-145 24884823 PMC4018186

[B14] Ayaz M. Junaid M. Ullah F. Sadiq A. Khan M. A. Ahmad W. (2015). Comparative chemical profiling, cholinesterase inhibitions and anti-radicals properties of essential oils from Polygonum hydropiper L: a preliminary anti-Alzheimer's study. Lipids Health Dis. 14:141 10.1186/s12944-015-0145-8 26530857 PMC4632677

[B15] Ayaz M. Sadiq A. Junaid M. Ullah F. Subhan F. Ahmed J. (2017). Neuroprotective and anti-aging potentials of essential oils from aromatic and medicinal plants. Front. Aging Neurosci. 9:168. 10.3389/fnagi.2017.00168 28611658 PMC5447774

[B16] Bahloul N. Bellili S. Aazza S. Chérif A. Faleiro M. L. Antunes M. D.. (2016). Aqueous extracts from tunisian diplotaxis: phenol content, antioxidant and anti-acetylcholinesterase activities, and impact of exposure to simulated gastrointestinal fluids. Antioxidants 5:12. 10.3390/antiox5020012 27049399 PMC4931533

[B17] Bartus R. T. (2000). On neurodegenerative diseases, models, and treatment strategies: lessons learned and lessons forgotten a generation following the cholinergic hypothesis. Exp. Neurol. 163, 495–529. 10.1006/exnr.2000.7397 10833325

[B18] Bartus R. T. Dean R. L. Beer B. Lippa A. S. (1982). The cholinergic hypothesis of geriatric memory dysfunction. Science 217, 408–414. 10.1126/science.7046051 7046051

[B19] Berkov S. Codina C. Viladomat F. Bastida J. (2008). N-Alkylated galanthamine derivatives: potent acetylcholinesterase inhibitors from Leucojum aestivum. Bioorg. Med. Chem. Lett. 18, 2263–2266. 10.1016/j.bmcl.2008.03.008 18356045

[B20] Besbes Hlila M. Omri A. Ben Jannet H. Lamari A. Aouni M. Selmi B. (2013). Phenolic composition, antioxidant and anti-acetylcholinesterase activities of the Tunisian Scabiosa arenaria. Pharmaceutical. Biol. 51, 525–532. 10.3109/13880209.2012.746713 23368937

[B21] Blanco-Silvente L. Castells X. Saez M. Barcelo M. A. Garre-Olmo J. Vilalta-Franch J.. (2017). Discontinuation, efficacy, and safety of cholinesterase inhibitors for Alzheimer's disease: a meta-analysis and meta-regression of 43 randomized clinical trials enrolling 16 106 patients. Int. J. Neuropsychopharmacol. 20, 519–528. 10.1093/ijnp/pyx012 28201726 PMC5492783

[B22] Bonesi M. Loizzo M. R. Conforti F. Passalacqua N. G. Saab A. Menichini F.. (2013). Berberis aetnensis and B. *libanotica:* a comparative study on the chemical composition, inhibitory effect on key enzymes linked to Alzheimer's disease and antioxidant activity. J. Pharmacy Pharmacol. 65, 1726–1735. 10.1111/jphp.12172 24236982

[B23] Bostan H. B. Mehri S. Hosseinzadeh H. (2017). Toxicology effects of saffron and its constituents: a review. Iran J. Basic Med. Sci. 20, 110–121. 10.22038/ijbms.2017.8230 28293386 PMC5339650

[B24] Cardoso-Lopes E. M. Maier J. A. Silva M. R. D. Regasini L. O. Simote S. Y. Lopes N. P.. (2010). Alkaloids from stems of *Esenbeckia leiocarpa* Engl. (Rutaceae) as potential treatment for Alzheimer disease. Molecules 15, 9205–9213. 10.3390/molecules15129205 21160449 PMC6259197

[B25] Colovic M. B. Krstic D. Z. Lazarevic-Pasti T. D. Bondzic A. M. Vasic V. M. (2013). Acetylcholinesterase inhibitors: pharmacology and toxicology. Curr. Neuropharmacol. 11, 315–335. 10.2174/1570159X11311030006 24179466 PMC3648782

[B26] Conforti F. Rigano D. Formisano C. Bruno M. Loizzo M. R. Menichini F. (2010). Metabolite profile and *in vitro* activities of *Phagnalon saxatile* (L.) Cass. relevant to treatment of Alzheimer's disease. J. Enz. Inhibit. Med. Chem. 25, 97–104. 10.3109/14756360903018260 20030514

[B27] Cortes N. Posada-Duque R. A. Alvarez R. Alzate F. Berkov S. Cardona-Gómez G. P.. (2015). Neuroprotective activity and acetylcholinesterase inhibition of five Amaryllidaceae species: a comparative study. Life Sci. 122, 42–50. 10.1016/j.lfs.2014.12.011 25529145

[B28] Craig L. A. Hong N. S. Mcdonald R. J. (2011). Revisiting the cholinergic hypothesis in the development of Alzheimer's disease. Neurosci. Biobehav. Rev. 35, 1397–1409. 10.1016/j.neubiorev.2011.03.001 21392524

[B29] Damar U. Gersner R. Johnstone J. T. Schachter S. Rotenberg A. (2016). Huperzine A as a neuroprotective and antiepileptic drug: a review of preclinical research. Expert Rev. Neurother. 16, 671–680. 10.1080/14737175.2016.1175303 27086593

[B30] Dastmalchi K. Ollilainen V. Lackman P. Af Gennäs G. B. Dorman H. D. Järvinen P. P.. (2009). Acetylcholinesterase inhibitory guided fractionation of *Melissa officinalis* L. Bioorg. Med. Chem. 17, 867–871. 10.1016/j.bmc.2008.11.034 19070498

[B31] Dvir H. Silman I. Harel M. Rosenberry T. L. Sussman J. L. (2010). Acetylcholinesterase: from 3D structure to function. Chem. Biol. Interact. 187, 10–22. 10.1016/j.cbi.2010.01.042 20138030 PMC2894301

[B32] Ellman G. L. Courtney K. D. Andres V. Jr Featherstone R. M. (1961). A new and rapid colorimetric determination of acetylcholinesterase activity. Biochem. Pharmacol. 7, 88–95. 10.1016/0006-2952(61)90145-9 13726518

[B33] Ferreres F. Grosso C. Gil-Izquierdo A. Valentão P. Andrade P. B. (2013). Phenolic compounds from Jacaranda caroba (Vell.) A. DC.: approaches to neurodegenerative disorders. Food Chem. Toxicol. 57, 91–98. 10.1016/j.fct.2013.03.012 23524314

[B34] Fiest K. M. Roberts J. I. Maxwell C. J. Hogan D. B. Smith E. E. Frolkis A.. (2016). The prevalence and incidence of dementia due to Alzheimer's disease: a systematic review and meta-analysis. Can. J. Neurol. Sci. 43, S51–S82. 10.1017/cjn.2016.36 27307128

[B35] Godyn J. Jonczyk J. Panek D. Malawska B. (2016). Therapeutic strategies for Alzheimer's disease in clinical trials. Pharmacol. Rep. 68, 127–138. 10.1016/j.pharep.2015.07.006 26721364

[B36] Greenblatt H. M. Kryger G. Lewis T. Silman I. Sussman J. L. (1999). Structure of acetylcholinesterase complexed with (-)-galanthamine at 2.3 A resolution. FEBS Lett. 463, 321–326. 10.1016/S0014-5793(99)01637-3 10606746

[B37] Gulcan H. O. Orhan I. E. Sener B. (2015). Chemical and molecular aspects on interactions of galanthamine and its derivatives with cholinesterases. Curr. Pharm. Biotechnol. 16, 252–258. 10.2174/1389201015666141202105105 25483718

[B38] Ha G. T. Wong R. K. Zhang Y. (2011). Huperzine a as potential treatment of Alzheimer's disease: an assessment on chemistry, pharmacology, and clinical studies. Chem. Biodivers. 8, 1189–1204. 10.1002/cbdv.201000269 21766442

[B39] Hamidpour M. Hamidpour R. Hamidpour S. Shahlari M. (2014). Chemistry, pharmacology, and medicinal property of sage (Salvia) to prevent and cure illnesses such as obesity, diabetes, depression, dementia, lupus, autism, heart disease, and cancer. J. Tradit. Complement. Med. 4, 82–88. 10.4103/2225-4110.130373 24860730 PMC4003706

[B40] Heinrich M. (2010). Galanthamine from galanthus and other amaryllidaceae–chemistry and biology based on traditional use. Alkaloids Chem. Biol. 68, 157–165. 10.1016/S1099-4831(10)06804-5 20334038

[B41] Henstridge C. M. Pickett E. Spires-Jones T. L. (2016). Synaptic pathology: a shared mechanism in neurological disease. Ageing Res. Rev. 28, 72–84. 10.1016/j.arr.2016.04.005 27108053

[B42] Holas O. Musilek K. Pohanka M. Kuca K. (2012). The progress in the cholinesterase quantification methods. Expert Opin. Drug Discov. 7, 1207–1223. 10.1517/17460441.2012.729037 23013366

[B43] Hostettmann K. Borloz A. Urbain A. Marston A. (2006). Natural product inhibitors of acetylcholinesterase. Curr. Org. Chem. 10, 825–847. 10.2174/138527206776894410

[B44] Houghton P. J. Ren Y. Howes M.-J. (2006). Acetylcholinesterase inhibitors from plants and fungi. Nat. Product Rep. 23, 181–199. 10.1039/b508966m 16572227

[B45] Huang L. Su T. Li X. (2013). Natural products as sources of new lead compounds for the treatment of Alzheimer's disease. Curr. Top. Med. Chem. 13, 1864–1878. 10.2174/15680266113139990142 23931437

[B46] Huang Q.-Q. Bi J.-L. Sun Q.-Y. Yang F.-M. Wang Y.-H. Tang G.-H.. (2012). Bioactive isoquinoline alkaloids from Corydalis saxicola. Planta Med. 78, 65–70. 10.1055/s-0031-1280126 21858757

[B47] Huq A. K. Jamal J. A. Stanslas J. (2014). Ethnobotanical, phytochemical, pharmacological, and toxicological aspects of persicaria hydropiper (l.) delarbre. Evid. Based Complement. Alternat. Med. 2014:782830 10.1155/2014/782830 24834098 PMC4009190

[B48] Imenshahidi M. Hosseinzadeh H. (2016). Berberis vulgaris and berberine: an update review. Phytother. Res. 30, 1745–1764. 10.1002/ptr.5693 27528198

[B49] Ingkaninan K. Temkitthawon P. Chuenchom K. Yuyaem T. Thongnoi W. (2003). Screening for acetylcholinesterase inhibitory activity in plants used in Thai traditional rejuvenating and neurotonic remedies. J. Ethnopharmacol. 89, 261–264. 10.1016/j.jep.2003.08.008 14611889

[B50] Johnson G. Moore S. W. (2006). The peripheral anionic site of acetylcholinesterase: structure, functions and potential role in rational drug design. Curr. Pharm. Des. 12, 217–225. 10.2174/138161206775193127 16454738

[B51] Jung H. A. Ali M. Y. Jung H. J. Jeong H. O. Chung H. Y. Choi J. S. (2016). Inhibitory activities of major anthraquinones and other constituents from *Cassia obtusifolia* against β-secretase and cholinesterases. J. Ethnopharmacol. 191, 152–160. 10.1016/j.jep.2016.06.037 27321278

[B52] Jung H. A. Karki S. Kim J. H. Choi J. S. (2015). BACE1 and cholinesterase inhibitory activities of Nelumbo nucifera embryos. Arch. Pharm. Res. 38, 1178–1187. 10.1007/s12272-014-0492-4 25300425

[B53] Jung H. A. Min B.-S. Yokozawa T. Lee J.-H. Kim Y. S. Choi J. S. (2009). Anti-Alzheimer and antioxidant activities of Coptidis Rhizoma alkaloids. Biol. Pharm. Bull. 32, 1433–1438. 10.1248/bpb.32.1433 19652386

[B54] Kamal Z. Ullah F. Ayaz M. Sadiq A. Ahmad S. Zeb A. (2015). Anticholinesterse and antioxidant investigations of crude extracts, subsequent fractions, saponins and flavonoids of *Atriplex laciniata* L.: potential effectiveness in Alzheimer's and other neurological disorders. Biol. Res. 48:21 10.1186/s40659-015-0011-1 25889712 PMC4393635

[B55] Kang S. Y. Lee K. Y. Sung S. H. Park M. J. Kim Y. C. (2001). Coumarins isolated from Angelica gigas inhibit acetylcholinesterase: structure-activity relationships. J. Nat. Prod. 64, 683–685. 10.1021/np000441w 11374978

[B56] Kennedy D. O. Scholey A. B. (2006). The psychopharmacology of European herbs with cognition-enhancing properties. Curr. Pharm. Design 12, 4613–4623. 10.2174/138161206779010387 17168769

[B57] Khan H. Amin S. Kamal M. A. Patel S. (2018). Flavonoids as acetylcholinesterase inhibitors: current therapeutic standing and future prospects. Biomed. Pharmacother. 101, 860–870. 10.1016/j.biopha.2018.03.007 29635895

[B58] Kuk E. B. Jo A. R. Oh S. I. Sohn H. S. Seong S. H. Roy A.. (2017). Anti-Alzheimer's disease activity of compounds from the root bark of *Morus alba* L. Arch. Pharm. Res. 40, 338–349. 10.1007/s12272-017-0891-4 28093699

[B59] Lai D.-H. Yang Z.-D. Xue W.-W. Sheng J. Shi Y. Yao X.-J. (2013). Isolation, characterization and acetylcholinesterase inhibitory activity of alkaloids from roots of Stemona sessilifolia. Fitoterapia 89, 257–264. 10.1016/j.fitote.2013.06.010 23831460

[B60] Lin H. Q. Ho M. T. Lau L. S. Wong K. K. Shaw P. C. Wan D. C. (2008). Anti-acetylcholinesterase activities of traditional chinese medicine for treating Alzheimer's disease. Chem. Biol. Interact. 175, 352–354. 10.1016/j.cbi.2008.05.030 18573242

[B61] Liu J.-S. Zhu Y.-L. Yu C.-M. Zhou Y.-Z. Han Y.-Y. Wu F.-W. (1986). The structures of huperzine A and B, two new alkaloids exhibiting marked anticholinesterase activity. Can. J. Chem. 64, 837–839. 10.1139/v86-137

[B62] Loizzo M. R. Menichini F. Tundis R. Bonesi M. Conforti F. Nadjafi F.. (2009). *In vitro* biological activity of Salvia leriifolia Benth essential oil relevant to the treatment of Alzheimer's disease. J. Oleo Sci. 58, 443–446. 10.5650/jos.58.443 19584571

[B63] Loizzo M. R. Tundis R. Conforti F. Menichini F. Bonesi M. Nadjafi F.. (2010). *Salvia leriifolia* Benth (Lamiaceae) extract demonstrates *in vitro* antioxidant properties and cholinesterase inhibitory activity. Nutri. Res. 30, 823–830. 10.1016/j.nutres.2010.09.016 21147365

[B64] Lopez S. Bastida J. Viladomat F. Codina C. (2002). Acetylcholinesterase inhibitory activity of some Amaryllidaceae alkaloids and Narcissus extracts. Life Sci. 71, 2521–2529. 10.1016/S0024-3205(02)02034-9 12270757

[B65] Ma X. Tan C. Zhu D. Gang D. R. Xiao P. (2007). Huperzine A from Huperzia species—an ethnopharmacolgical review. J. Ethnopharmacol. 113, 15–34. 10.1016/j.jep.2007.05.030 17644292

[B66] Madeo J. Elsayad C. (2013). The role of oxidative stress in Alzheimer's disease. J. Alzheimers Dis. Parkinsonism 3, 116–121. 10.4172/2161-0460.1000116

[B67] Mehta M. Adem A. Sabbagh M. (2012). New acetylcholinesterase inhibitors for Alzheimer's disease. Int. J. Alzheimers Dis. 2012:728983 10.1155/2012/728983 22216416 PMC3246720

[B68] Murray A. P. Faraoni M. B. Castro M. J. Alza N. P. Cavallaro V. (2013). Natural AChE inhibitors from plants and their contribution to Alzheimer's disease therapy. Curr. Neuropharmacol. 11, 388–413. 10.2174/1570159X11311040004 24381530 PMC3744903

[B69] Nwidu L. L. Elmorsy E. Thornton J. Wijamunige B. Wijesekara A. Tarbox R.. (2017). Anti-acetylcholinesterase activity and antioxidant properties of extracts and fractions of Carpolobia lutea. Pharm. Biol. 55, 1875–1883. 10.1080/13880209.2017.1339283 28629287 PMC6130458

[B70] Ohba T. Yoshino Y. Ishisaka M. Abe N. Tsuruma K. Shimazawa M.. (2015). Japanese Huperzia serrata extract and the constituent, huperzine A, ameliorate the scopolamine-induced cognitive impairment in mice. Biosci. Biotechnol. Biochem. 79, 1838–1844. 10.1080/09168451.2015.1052773 26059088

[B71] Orhan I. E. Orhan G. Gurkas E. (2011). An overview on natural cholinesterase inhibitors–a multi-targeted drug class–and their mass production. Mini. Rev. Med. Chem. 11, 836–842. 10.2174/138955711796575434 21762104

[B72] Ovais M. Ayaz M. Khalil A. T. Shah S. A. Jan M. S. Raza A. (2018). HPLC-DAD finger printing, antioxidant, cholinesterase, and α-glucosidase inhibitory potentials of a novel plant Olax nana. BMC Complement. Alter. Med. 18:1 10.1186/s12906-017-2057-9 PMC5751879 29295712

[B73] Penumala M. Zinka R. B. Shaik J. B. Amooru Gangaiah D. (2017). *In vitro* Screening of three indian medicinal plants for their phytochemicals, anticholinesterase, antiglucosidase, antioxidant, and neuroprotective effects. Biomed. Res. Int. 2017:5140506 10.1155/2017/5140506 29204442 PMC5674485

[B74] Penumala M. Zinka R. B. Shaik J. B. Mallepalli S. K. R. Vadde R. Amooru D. G. (2018). Phytochemical profiling and *in vitro* screening for anticholinesterase, antioxidant, antiglucosidase and neuroprotective effect of three traditional medicinal plants for Alzheimer's disease and diabetes mellitus dual therapy. BMC Complement. Alter. Med. 18:77 10.1186/s12906-018-2140-x PMC5834903 29499679

[B75] Pereira D. M. Ferreres F. Oliveira J. M. Gaspar L. Faria J. Valentão P.. (2010). Pharmacological effects of Catharanthus roseus root alkaloids in acetylcholinesterase inhibition and cholinergic neurotransmission. Phytomedicine 17, 646–652. 10.1016/j.phymed.2009.10.008 19962870

[B76] Perry N. S. Bollen C. Perry E. K. Ballard C. (2003). Salvia for dementia therapy: review of pharmacological activity and pilot tolerability clinical trial. Pharmacol. Biochem. Behav. 75, 651–659. 10.1016/S0091-3057(03)00108-4 12895683

[B77] Prince M. J. (2015). World Alzheimer Report 2015: The Global Impact of Dementia: An Analysis of Prevalence, Incidence, Cost and Trends. London: Alzheimer's Disease International.

[B78] Pueyo I. U. Calvo M. I. (2009). Assay conditions and validation of a new UV spectrophotometric method using microplates for the determination of polyphenol content. Fitoterapia 80, 465–467. 10.1016/j.fitote.2009.06.008 19540907

[B79] Qian Z. M. Ke Y. (2014). Huperzine A: is it an effective disease-modifying drug for Alzheimer's disease? Front. Aging Neurosci. 6:216. 10.3389/fnagi.2014.00216 25191267 PMC4137276

[B80] Rafii M. Walsh S. Little J. Behan K. Reynolds B. Ward C.. (2011). A phase II trial of huperzine A in mild to moderate Alzheimer disease. Neurology 76, 1389–1394. 10.1212/WNL.0b013e318216eb7b 21502597 PMC3269774

[B81] Rasheed D. M. El Zalabani S. M. Koheil M. A. El-Hefnawy H. M. Farag M. A. (2013). Metabolite profiling driven analysis of Salsola species and their anti-acetylcholinesterase potential. Nat. Prod. Res. 27, 2320–2327. 10.1080/14786419.2013.832676 24028607

[B82] Reza A. A. Hossain M. S. Akhter S. Rahman M. R. Nasrin M. S. Uddin M. J. (2018). *In vitro* antioxidant and cholinesterase inhibitory activities of Elatostema papillosum leaves and correlation with their phytochemical profiles: a study relevant to the treatment of Alzheimer's disease. BMC Complement. Alter. Med. 18:123 10.1186/s12906-018-2182-0 PMC5887180 29622019

[B83] Rollinger J. M. Hornick A. Langer T. Stuppner H. Prast H. (2004). Acetylcholinesterase inhibitory activity of scopolin and scopoletin discovered by virtual screening of natural products. J. Med. Chem. 47, 6248–6254. 10.1021/jm049655r 15566295

[B84] Sahoo A. K. Dandapat J. Dash U. C. Kanhar S. (2018). Features and outcomes of drugs for combination therapy as multi-targets strategy to combat Alzheimer's disease. J. Ethnopharmacol. 215, 42–73. 10.1016/j.jep.2017.12.015 29248451

[B85] Saleem H. Ahmad I. Shahid M. N. Gill M. Nadeem M. F. Mahmood W.. (2016). *In vitro* acetylcholinesterase and butyrylcholinesterase inhibitory potentials of Jatropha gossypifolia plant extracts. Acta Poloniae Pharmaceut. Drug Res. 73, 419–423. 27180434

[B86] Sarker S. D. Latif Z. Gray A. I. (2005). Natural Products Isolation. Totowa, NJ: Humana Press.

[B87] Scheltens P. Blennow K. Breteler M. M. De Strooper B. Frisoni G. B. Salloway S.. (2016). Alzheimer's disease. Lancet 388, 505–517. 10.1016/S0140-6736(15)01124-1 26921134

[B88] Schrattenholz A. Pereira E. F. Roth U. Weber K. H. Albuquerque E. X. Maelicke A. (1996). Agonist responses of neuronal nicotinic acetylcholine receptors are potentiated by a novel class of allosterically acting ligands. Mol. Pharm. 49, 1–6. 8569694

[B89] Shu Y.-Z. (1998). Recent natural products based drug development: a pharmaceutical industry perspective. J. Nat. Prod. 61, 1053–1071. 10.1021/np9800102 9722499

[B90] Suganthy N. Pandian S. K. Devi K. P. (2009). Cholinesterase inhibitory effects of rhizophora lamarckii, avicennia officinalis, sesuvium portulacastrum and suaeda monica: mangroves inhabiting an Indian coastal area (Vellar Estuary). J. Enz. Inhibit. Med. Chem. 24, 702–707. 10.1080/14756360802334719 18686140

[B91] Takizawa C. Thompson P. L. Van Walsem A. Faure C. Maier W. C. (2015). Epidemiological and economic burden of Alzheimer's disease: a systematic literature review of data across Europe and the United States of America. J. Alzheimers Dis. 43, 1271–1284. 10.3233/JAD-141134 25159675

[B92] Tarawneh R. Holtzman D. M. (2012). The clinical problem of symptomatic Alzheimer disease and mild cognitive impairment. Cold Spring Harbor Perspect. Med. 2:a006148. 10.1101/cshperspect.a006148 22553492 PMC3331682

[B93] Thomsen T. Kewitz H. (1990). Selective inhibition of human acetylcholinesterase by galanthamine *in vitro* and *in vivo*. Life Sci. 46, 1553–1558. 10.1016/0024-3205(90)90429-U 2355800

[B94] Tune L. E. (2001). Anticholinergic effects of medication in elderly patients. J. Clin. Psychiatry 62, 11–14. 11584981

[B95] Tung B. T. Hai N. T. Thu D. K. (2017). Antioxidant and acetylcholinesterase inhibitory activities *in vitro* of different fraction of *Huperzia* squarrosa (Forst.) Trevis extract and attenuation of scopolamine-induced cognitive impairment in mice. J. Ethnopharmacol. 198, 24–32. 10.1016/j.jep.2016.12.037 28025162

[B96] Uriarte-Pueyo I. Calvo M. (2011). Flavonoids as acetylcholinesterase inhibitors. Curr. Med. Chem. 18, 5289–5302. 10.2174/092986711798184325 22087826

[B97] Wong K. K.-K. Ho M. T.-W. Lin H. Q. Lau K.-F. Rudd J. A. Chung R. C.-K.. (2010). Cryptotanshinone, an acetylcholinesterase inhibitor from *Salvia miltiorrhiza*, ameliorates scopolamine-induced amnesia in Morris water maze task. Planta Med. 76, 228–234. 10.1055/s-0029-1186084 19774505

[B98] Yang Z.-D. Zhang X. Du J. Ma Z.-J. Guo F. Li S.. (2012). An aporphine alkaloid from *Nelumbo nucifera* as an acetylcholinesterase inhibitor and the primary investigation for structure–activity correlations. Nat. Prod Res. 26, 387–392. 10.1080/14786419.2010.487188 21732870

[B99] Zhao Y. Dou J. Wu T. Aisa H. A. (2013). Investigating the antioxidant and acetylcholinesterase inhibition activities of *Gossypium herbaceam*. Molecules 18, 951–962. 10.3390/molecules18010951 23344203 PMC6269909

